# Creating a Community-Based Medical Clinic Dedicated to Patients Experiencing Homelessness in New York City (NYC): The Weill Cornell Street Medicine Clinic

**DOI:** 10.7759/cureus.81797

**Published:** 2025-04-06

**Authors:** Ashwin Mahesh, Barr Lavi-Romer, Amanda Ramsdell

**Affiliations:** 1 Hospital Medicine, Weill Cornell Medicine, New York City, USA

**Keywords:** community health, free clinic, housing, public health, street medicine

## Abstract

Patients experiencing homelessness (PEH) continue to experience acute and chronic medical vulnerabilities in the United States. In the acute setting, these disparities increase hospital readmission rates, utilization of optimal treatment, and mortality. In the chronic setting, these disparities increase housing instability, medical deterioration, and mortality. These vulnerabilities are exacerbated in New York City (NYC), with PEH experiencing longer hospital stays, increased hospitalization costs, and disproportionate mental health and substance use hospitalizations. Over the past few decades, community interventions delivering medical care directly to PEH, in unison with social support structures, have shown improvements in both medical and housing-based outcomes. Here, we describe the step-wise approach we used in founding the Weill Cornell Street Medicine Clinic, a free, community-based medical clinic dedicated to serving the medical and social needs of PEH in NYC. We walk through our processes for conducting needs assessments, community partner selection, stakeholder recruitment, acquiring funding, establishing a robust legal framework, creating electronic medical record infrastructure, developing free prescription and transportation programs, establishing a free laboratory partnership, and reflections from our first year of operations. Through this report, we hope to provide a reproducible methodology to support the implementation of similar programs serving PEH in communities across the United States.

## Introduction

The relationship between healthcare and housing is complex, bidirectional, and often a primary driver in the initiation and perpetuation of cycles of chronic homelessness. For many patients experiencing homelessness (PEH), a lack of access to stable and consistent healthcare leads to both acute and chronic vulnerability. Disparities in medical care for acute health concerns lead to worsened medical outcomes for PEH, including mortality. One study that investigated disparities in cardiovascular outcomes for PEH found that, in the acute setting, PEH had higher mortality for ST-elevation myocardial infarctions, stroke, and cardiac arrest when compared to patients not experiencing homelessness [1]. Another study investigating outcomes for PEH hospitalized for gastrointestinal bleeding found that PEH had significantly increased mortality when compared to patients not experiencing homelessness, with both higher 30-day readmission rates and lower rates of endoscopy utilization [2]. Many reasons have been proposed for this increased acute medical vulnerability that PEH face, including barriers to building trust, medical provider bias, and a lack of available, robust systems to establish and maintain close medical follow-up with communities experiencing homelessness.

The disparities at the intersection of medical care and housing not only create acute vulnerabilities, but also foster chronic vulnerability. With respect to the management of chronic medical conditions, PEH, on average, receive fewer preventative screenings and interventions. For instance, in the management of diabetes, experiencing homelessness is associated with less frequent A1C testing, serum creatinine testing, urine protein assessment, and ophthalmologic evaluations. Furthermore, PEH are less likely to utilize primary care or specialist care for diabetes management [3]. Additional studies have indicated that PEH are more likely to have uncontrolled hypertension than patients with stable housing [4]. Of note, the prevalence of smoking is significantly higher in many populations experiencing homelessness, due to a variety of factors, including increased stressors and lower access to cessation resources. One study in 2016 estimated that approximately 73% of adults experiencing homelessness smoke cigarettes, compared to general population estimates at the time of approximately 15.5% [5]. Notably, this increased smoking prevalence has been hypothesized to play a role in driving the elevated rates of obstructive lung disease in populations experiencing homelessness, estimated to be double the general population [6]. Together, these noted health disparities in diabetes, hypertension, and smoking use have been theorized to account for a significant portion of the tripled risk of cardiovascular disease in PEH and increased mortality compared to the general population [4]. PEH also have a significantly higher estimated lifetime prevalence of mental health conditions as compared to the general population (77% [7] vs. 29.2% [8]). Furthermore, PEH face significant barriers to accessing mental health care, including longitudinal prescription and provider coverage, worsening chronic vulnerability. Many PEH cite these barriers to mental health care access as a key factor increasing the difficulty of establishing stable housing [9]. Without targeted healthcare interventions dedicated to establishing support for these acute and chronic vulnerabilities, PEH are at risk of continued medical deterioration, housing instability, and mortality.

As evidenced above, healthcare landscapes for PEH in the United States have centered around the management of late-stage chronic illnesses, rather than primary prevention and early-stage intervention. Studies have demonstrated that PEH have significantly lower utilization of primary care [10], and increased emergency department utilization [11], compared to housed counterparts. Over the past few decades, innovative community-based interventions delivering healthcare directly within the context of the lives of PEH, and in collaboration with social support services, have demonstrated improvements in not only acute and chronic health outcomes, but also housing-centered outcomes [12-16]. Despite vast diversity in intervention implementation, strategy, and healthcare delivery, common developmental features and improvements can be observed and subsequently replicated to better meet the needs of PEH across the United States. 

Specifically, there is an established urgent need for the development of evidence-based community healthcare interventions serving PEH in New York City (NYC). There are an estimated 350,000 individuals without stable housing living in NYC as of 2024, with a majority of those who are unsheltered living with serious health conditions [17]. Without early, upstream intervention, the vast and rapidly expanding population experiencing homelessness in NYC is at significant risk for worsening medical outcomes, with increased emergency department utilization [18], longer hospitalization stays, increased hospitalization costs, and disproportionate mental health and substance use hospitalizations [19].

With these clear disparities in mind, here we describe an evidence-based approach that we utilized to launch the Weill Cornell Street Medicine Clinic, a community-based medical clinic embedded within the New York Common Pantry (NYCP) in East Harlem, dedicated to serving the medical and social needs of PEH in NYC. Throughout the creation of our clinic, we followed core principles of street medicine, the practice of delivering medical care directly to PEH within the contexts of their lives. Our goal in this paper is to describe a stepwise approach to launching community-based medical clinics for PEH, strategies for stakeholder recruitment and engagement in large health systems, replicable methodology for creating supportive care services, challenges encountered, unique considerations in NYC, and future goals in development. By doing so, we hope to provide reproducible strategies to support the creation and implementation of similar programs to serve communities experiencing homelessness in health systems across the United States.

## Technical report

Mission and purpose

The Weill Cornell Street Medicine Clinic was founded with a core mission of elevating the voices of PEH in NYC and partnering together to drive durable change in disparities at the intersection of housing and healthcare. 

Many PEH have faced years of neglect, abuse, and trauma within health systems in the United States. Recognizing this, one of our primary goals was to reestablish longitudinal trust with communities experiencing homelessness and utilize this trust to help increase both health and housing outcomes for our patients. With growing trust, we then aimed to establish an approachable, safe, and fully free medical clinic for PEH to access needed medical care directly within the contexts of their lives, and ingrained in systems of pre-established social care. In addition to core medical services, we established a goal of providing extensive medical and social support programs driven by the unmet needs of our community, including free prescription, laboratory, and transportation programs. We further prioritized developing an organizational structure rooted in continual expansion and improvement of services, centered around feedback from our patients and community partners. Under this aim, we set out to develop an extensive resource and referral connection network to anticipate patient needs. Initial measures of effectiveness for our clinic were established, including the ability to establish continuity of care for patients through our clinic or referrals, early detection and management of chronic illness, connection to social care resources and follow-through, and proportion of budget invested directly into patient services. Finally, we set an overarching goal of expanding care services beyond our clinic in order to support PEH across our health system.

Step 1: needs assessment

**Figure 1:**
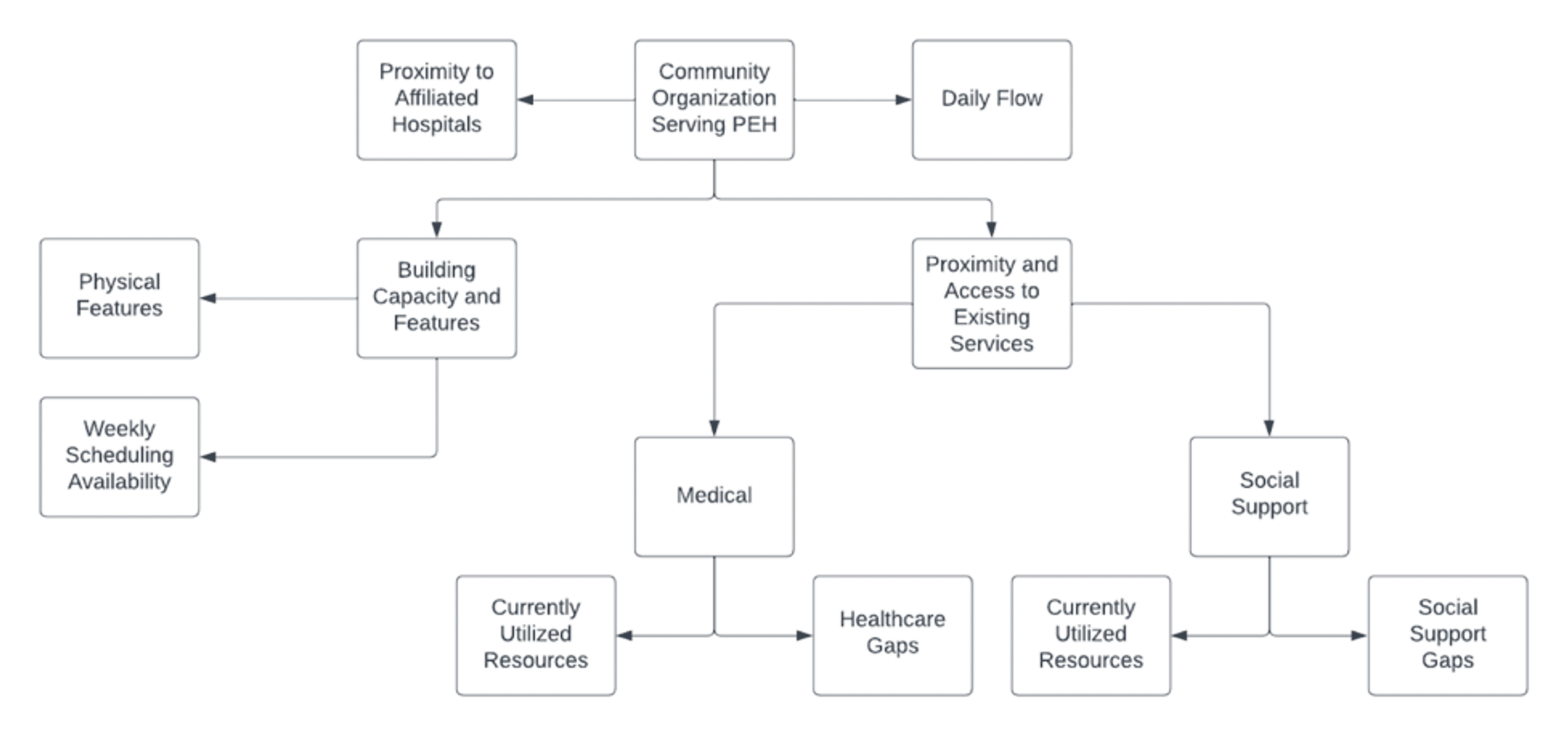
Community partner resource mapping developed for conducting systematic needs assessment PEH, Patients experiencing homelessness

The first step in establishing our clinic was to identify areas of need within NYC; specifically, we set out to identify communities of PEH with the greatest disparity in access to medical resources. Over the course of several months, we volunteered with and systematically evaluated 12 different community partners serving individuals experiencing homelessness across Manhattan. We developed resource maps for each community partner, specifically focused on key factors such as the daily flow of PEH, proximity and access to existing medical and social support services, building capacity and features, and proximity to Weill Cornell affiliated hospitals (Figure 1). With respect to daily flow, we split the analysis into “high-flow” and “low-flow,” defined respectively as more or less than 100 individuals accessing a site daily. We further characterized sites as “closed,” “hybrid,” or “open” systems based on accessibility; “closed” systems could be accessed by one constant group of individuals experiencing homelessness (i.e., closed shelter), “hybrid” were both accessible to a constant group and open to the public during certain hours, and “open” were accessible to the public for most services. With respect to proximity and access to existing medical services, we analyzed each site for the closest low-cost or free medical services and stratified by average wait times at each access point for establishing care. Where possible, we evaluated the extent to which available medical resources were known and being utilized by each community. We also looked at the diversity of medical resources present, either within or near each community partner, and identified key gaps in healthcare for PEH if present. With respect to proximity and access to social support services, we learned about each community partner, the social support network provided by each site, and opportunities for future collaboration. Specifically, we looked at the delivery of key programs targeting food insecurity, insurance connections, housing stability, and employment opportunities. This was of particular importance in supporting our core mission of building a multidisciplinary clinic rooted in systems of robust social care. With respect to building capacity and features, we evaluated each site for the presence of a private space for physicians to see patients and weekly scheduling availability. Finally, we looked at proximity to Weill Cornell affiliated hospitals in consideration of future development goals and the possibility of integration of our clinic services with hospital programs.

Based on our mission and longitudinal goals, we prioritized factors such as high patient flow, “open” systems, lack of access to robust medical care, presence of continued and varied social support services, the presence of private space and weekly scheduling ability, and closer proximity to Weill Cornell affiliated hospitals in our systematic analysis.

Step 2: community partner selection

In reviewing the results of our needs assessment evaluating community partners serving PEH across Manhattan, one site stood out as being particularly well-suited for the establishment of a partnership in line with our clinic’s intended goals. Importantly, our research indicated that this site, the NYCP, served a high flow of PEH consisting of 200 to 300 individuals daily, with services predominantly following an open system of care. Furthermore, the NYCP provided daily onsite case management for PEH, including access to public benefits, supportive housing assistance, psychiatric evaluations, supplemental security income applications, onsite mail services, birth certificate and state identification procurement, and extensive referral services. Of particular significance was the identification of the NYCP’s recent loss of previously existing onsite medical care, demonstrating an active need for a community partner providing medical care. The NYCP also had a history of over four decades of operation within East Harlem, indicating strong foundations of community trust, as well as a large enclosed physical space with weekly scheduling ability, allowing for private clinic visits without disruption of site operations. Additionally, the NYCP was located within three miles of Weill Cornell’s main hospital campus, with easy and direct access through public transit.

We established a line of communication with our community partner’s program managers and social workers, sharing our clinic’s mission and longitudinal goals, and inquiring about their perception of current needs and availability for partnership. With the support of the NYCP’s program managers, our team arranged a meeting with the site’s social workers in order to begin to better understand their assessment of current needs and confirm the utility of our clinic within their community. Upon confirming their belief in a role for our clinic within their community and interest in partnership establishment, we looked towards further stakeholder recruitment, funding acquisition, and legal infrastructure establishment, as detailed below.

Step 3: stakeholder recruitment

After our needs assessment and community partner selection, we developed a plan of action detailing the steps required to launch our clinic. An integral part of this plan was recruiting key stakeholders at each step to both advocate for improvements in our structure and establish longitudinal sustainability of processes. First and foremost, we recruited integral partners within Weill Cornell Medical College and the Office of Student Affairs for guidance on medical student participation and risk mitigation. Additionally, we recruited representation at Risk Management at Weill Cornell Medicine (WCM) to approve proposed physician clinical activities, as well as student involvement in clinical workflow. We further established a legal framework with various counsel across our health system, detailed further under Step 5. We then collaborated closely with the Office of Affiliations at WCM to officiate partnership with the NYCP and explicitly delineate patient protections and organizational responsibilities.

In addition, we recruited strategic funding and vision partners at the NYP Community and Population Department, described more in Step 4. In order to develop our electronic medical record (EMR) infrastructure at no cost to our clinic, we recruited the support of leaders within the Epic Development team at our health system (detailed in Step 6). For guidance on logistics surrounding billing suppression, we recruited executives managing the Revenue Cycle at the WCM Physician Organization (PO). Anticipating needs for continued low-cost medical equipment, we partnered directly with medical supply sourcing leaders at our health system to utilize beneficial, higher supply volume pricing for orders. For the development of our free laboratory services program, we recruited the involvement of executives of a large lab services provider, described more in Step 7. For advice on projections for supplementary transportation and prescription services, we recruited the support of our social work department at our health system and financial management for pre-existing charity care programs (further described under Step 7).

Moreover, we recruited a wide referral network for medical services, anticipating potential needs within our diverse patient population. This network of partnerships included options for alternative primary care follow-up sites for both uninsured and insured patients, clinics for patients with substance use conditions, free vision care for uninsured patients, and more.

Step 4: acquiring funding

In order to acquire funding to launch our clinic, we looked for both longitudinal and temporary funding opportunities through partnerships and grants. Using mathematical modeling, we established tailored projections for both the minimum our clinic required annually to support patients, and higher funding goals that would allow for expansion and growth to serve greater volumes of patients. Our initial projections focused on the domains of medical equipment, medical supplies, patient transportation, prescription assistance, laboratory fees, and IT infrastructure. Under medical equipment and supplies, we broke down projections into one-time fixed costs (i.e., durable medical equipment such as otoscopes and blood pressure monitors) and longitudinal recurrent costs (such as A1C kits, gloves, sanitizer, urine dipsticks, etc.). For medical appointment transportation, we estimated the costs of covering transportation for both patients that we see for clinic appointments, and additional patients that we are able to assist with health navigation while onsite. We established both conservative and liberal projections based on the average cost of a Lyft ride within Manhattan, and the costs of metro transportation. For prescription assistance, we leveraged estimations from our needs assessment of the proportion of uninsured and insured patients that access the NYCP each day to create projections of the approximate per-patient cost burden of prescriptions. We further stratified estimations by predicted prescribing frequency and compounded estimates of the average number of patients utilizing our program over time. One key limitation in our initial projections includes an unknown distribution of predominant health concerns within our patient population. With respect to laboratory fees, we similarly leveraged insurance proportion estimations from our needs assessment and utilized cost data on the most commonly sent lab orders, inclusive of draw, processing, and interpretation fees. Finally, for IT infrastructure fees, we projected the cost of building an EMR department with internal health system quotes.

Though we were able to obtain one-time grants to cover a portion of our projections, a majority of our funding came from partnerships within our health system. After projection to numerous departments, organizations, and individuals within our health system, we were able to partner with the NYP Community and Population Health Department to cover costs for medical equipment and supplies, patient transportation, and prescription assistance budgets. Through our strategic program development with an external laboratory provider (as discussed in Step 7), we were able to forgo costs projected for laboratory fees, and through partnership within our health system, we were able to have the cost of EMR development covered.

Step 5: legal infrastructure

In order to establish our clinic directly within the NYCP, a site not accredited or affiliated with our health system, we had to establish a framework for patient protection, physician liability, non-medical staff liability, and risk management. In order to build this framework, we collaborated with representation from the Legal Counsel from our PO, Risk Management from our institution, and, when applicable for services, Legal Counsel from our larger health system. Drafting of agreements for supplementary services and processes occurred through direct collaboration and advice from Legal Counsel from our PO, with subsequent input and signature from administrative leaders across our institution, dependent on the process and necessary clearance.

Step 6: EMR department creation

**Figure 2:**
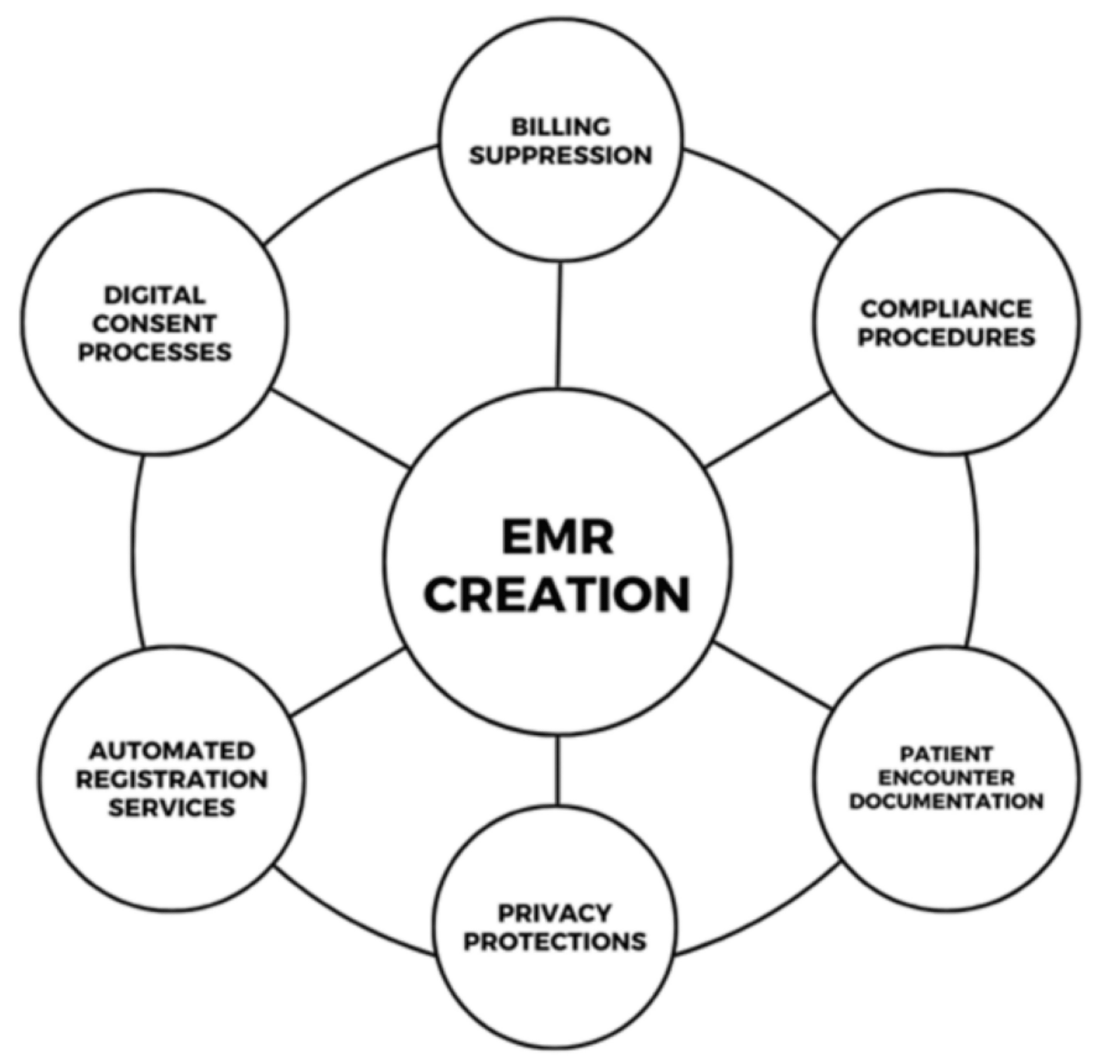
Key domains for EMR development for the Weill Cornell Street Medicine Clinic EMR, Electronic medical record

In order to provide a robust system of organization for our patients, safety in patient care mechanisms, and smooth integration with departments within our health system, we looked for ways to develop and customize an EMR department. We reached out to an Epic development team affiliated with our health system and collaborated over the course of one year to create a new Epic context for our clinic. Through the process of developing this department, we aimed to establish a fluid interface that mirrored the intended flexibility of our clinic workflows. Key domains that we focused on developing over the course of this year were the creation of digital consent processes, automated registration services, patient compliance procedures, patient privacy protections within a non-health system location, flexible workflows for patients with unique considerations (for example, no stable address), automated billing suppression and internal revenue cycle approval, encounter documentation procedures, and more (Figure 2). We were able to launch and test this EMR prior to our official clinic launch, to ensure the effectiveness of processes for use in patient care.

Step 7: development of supporting patient services

Based on our preliminary needs assessment with our community partner, we discovered an important need for the creation of specific support services to help reduce barriers to accessing needed medical care.

Prescription Assistance Program

**Figure 3:**
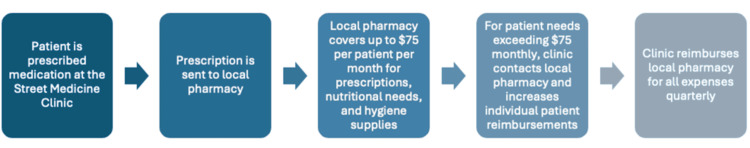
Prescription assistance program workflow with community pharmacy

We learned that a significant proportion of our patient population is uninsured or holds unstable insurance status, making continued access to needed medications difficult for many of our patients. Utilizing funding acquired from the NYP Community and Population Health Department, we developed a novel prescription assistance program, partnering with a local pharmacy near the NYCP to provide free and subsidized prescriptions for our patients, regardless of insurance status. Through this partnership, we drafted and executed a legal agreement between WCM and a local community pharmacy for our clinic to pay for up to $75 per month per patient for prescriptions, with the opportunity to increase coverage amount based on individual patient needs. In addition, we built a clause within this contract to help support patients with nutritional and hygiene needs, again budgeting up to $75 per month per patient, with the opportunity to increase based on individual circumstances. Our implementation and reimbursement strategy is described in Figure 3.

Free Laboratory Service Program

**Figure 4:**
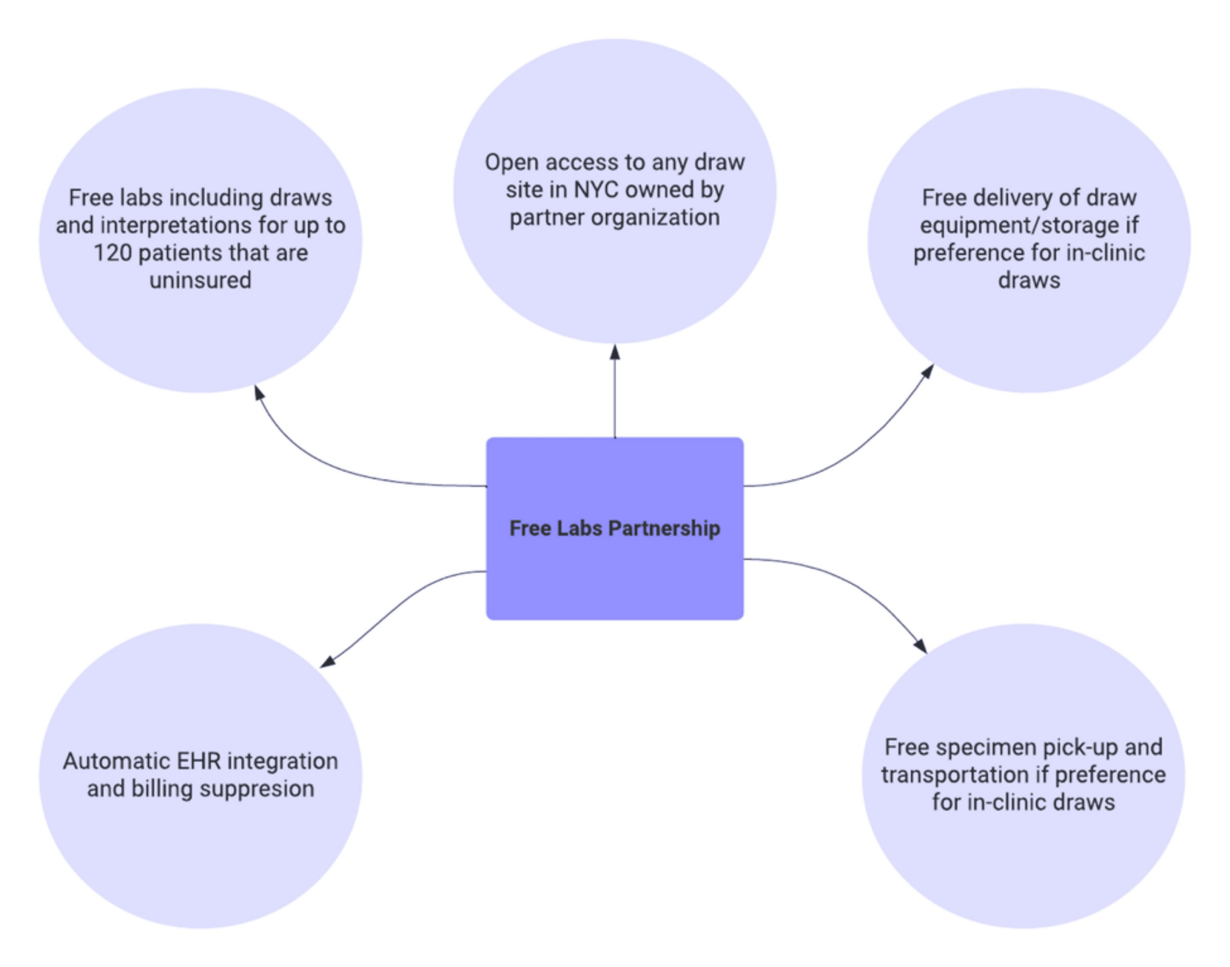
Components of free laboratory services agreement with a leading organization in diagnostic information services EHR, Electronic health record; NYC, New York City

Given the high cost of laboratory fees and interpretation, our clinic sought out connections with both internal and external organizations in order to mitigate expenses and maintain the ability to provide our patients with truly free care. Given that our initial projection for laboratory fees and interpretation was not able to be accommodated by our hospital’s internal laboratory, we began looking for reputable organizations to partner with for our patients. We were able to get in contact with a leading provider of diagnostic information services and began conversations to draft a novel agreement to provide free labs to uninsured patients accessing our clinic. Given that this was the first free contract that this organization had provided outside of federally qualified health centers in the Northeast region, we worked for approximately one year to establish EMR infrastructure compatibility and meet compliance requirements. Ultimately, our clinic was able to receive free labs and interpretations for up to 120 uninsured patients annually, with additional access to free lab draws for our patients at any of the many draw sites this organization has throughout NYC. More details about this agreement can be found in Figure 4.

Free Transportation Assistance Program

Transportation is a key barrier restricting access to care in patients experiencing poverty, often leading to delays in necessary care [20]. This barrier is exacerbated in NYC as a result of an increasingly complex medical landscape, as well as disparate access to timely transportation across the five boroughs. When paired with life circumstances and hardships that many PEH face, lack of stable transportation is often a key barrier to obtaining stable medical care.

With this in mind, we utilized funding acquired from the NYP Community and Population Health Department to develop a free transportation program to support our patients with getting to and from specialist and follow-up appointments. We partnered with Lyft (Lyft, Inc., San Francisco, CA, USA) to establish an account to schedule transportation for patients, and we purchased MetroCards in bulk for patients willing and able to utilize public transportation. This program supports the transportation needs of both patients coming in for health navigation and patients seen in the clinic by our physicians.

Summary of steps

**Figure 5:**
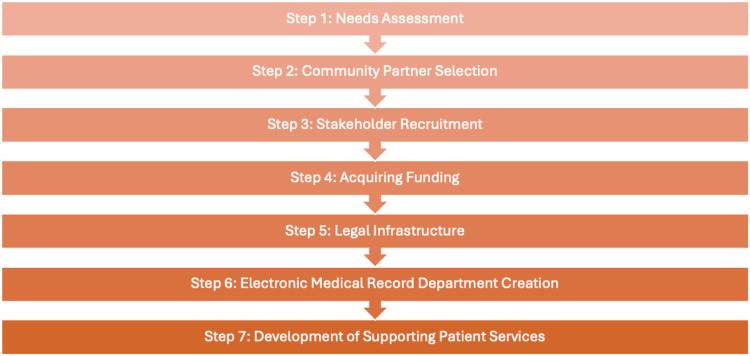
Summary of steps to launch the Weill Cornell Street Medicine Clinic

The steps outlined above to launch the Weill Cornell Street Medicine Clinic are summarized in Figure 5.

Launch and pilot interval impacts

The Weill Cornell Street Medicine Clinic officially launched in March of 2024, after almost two years of development and organization. Between March 2024 and January 2025, our clinic established a pilot interval, where we focused on evaluating the efficacy of clinic programs, supporting services, partner referrals, and more. During this interval, we held clinics at a monthly frequency and limited our patient capacity to three to four patients per clinic to ensure quality of care in our processes. Each care mechanism was closely monitored and evaluated to guarantee follow-through for our patients. As this pilot interval closed, starting in February of 2025, we doubled our physician staffing capacity and expanded our frequency to twice monthly. As we continue to develop, we intend to progressively expand both our clinic frequency and hours of operation to increase the number of patients we are able to serve. We are currently in the process of tracking patient follow-up rates and treatment outcomes and plan to analyze this data as sample size increases.

During our pilot interval, we were able to see 26 patients for official medical appointments with our physicians, conduct approximately 87 medical screening tests outside of appointments (including blood pressure and A1C evaluations), and place 17 successful medical and specialist referrals. We have also been able to integrate closely with social work at the NYCP, routinely helping patients establish health insurance, prescription coverage, transportation assistance, access employment resources, food security, and hygiene supplies. 

**Figure 6:**
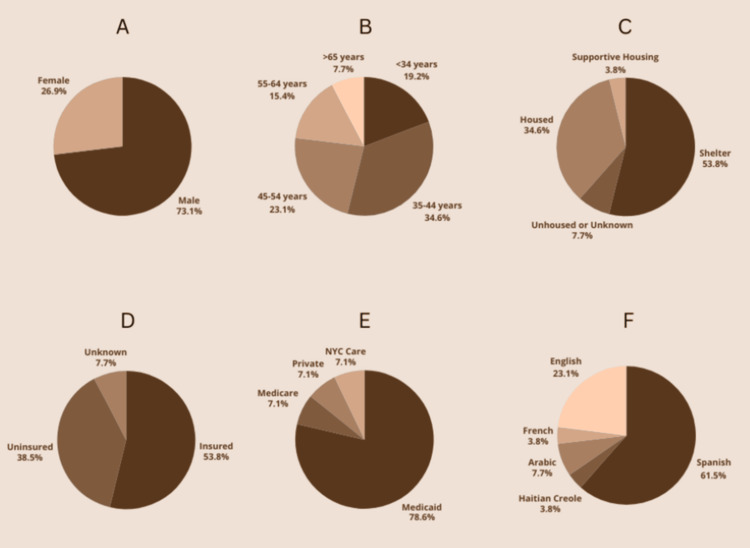
WCM Street Medicine Clinic patient population demographic information A) gender, B) age, C) housing, D) insurance status, E) insurance type, and F) primary language WCM, Weill Cornell Medicine; NYC, New York City

With respect to our patient population, 19 (73%) of our patients were male, 7 (27%) were female, 14 (54%) were under the age of 45, 15 (58%) noted living in shelters or being unhoused, 10 (38%) were uninsured, 11 (42%) had Medicaid, and 16 (62%) spoke Spanish as a primary language. Of note, data on unstable housing within housing status was not ascertained. More information can be found in Figure 6.

## Discussion

The establishment of community-based clinics is one strategy to counter the inaccessibility of high-quality healthcare to vulnerable populations, including PEH. Our experience launching one such clinic demonstrates some early successes and challenges associated with this approach.

One key factor we identified as essential to our clinic’s successful launch was its conception, rooted in community needs. By beginning the establishment of our clinic with a needs assessment evaluating community partners serving PEH in NYC, we were able to better understand the current disparities landscape, justify the creation of a clinic to potential partners, and ensure the development of a service centered around the local population.

Another key factor to our clinic’s success was the prioritization of our unique patient population in our clinic design. In selecting our first pharmacy partnership, we sought out a local pharmacy within four city blocks of our clinic, increasing ease of access. For those patients requiring non-local follow-up care, we established a comprehensive transportation assistance program, allowing for the scheduling of rides to and from appointments, along with free MetroCards for those patients preferring public transportation. These measures held significant weight in building patient trust and centering patient needs.

Ultimately, our experience demonstrated an opportunity for medical school and affiliated institutional resources to be diverted directly to PEH; however, the process of doing so required early stakeholder investment, persistence, and a solutions-oriented perspective. While our clinic was designed so that all clinical care would be physician-delivered, the establishment of the clinic itself required more time than most working physicians could access, making medical students one key group with sufficient time, energy, and clinical connections to tackle the process of establishing a community-based medical clinic.

Preliminary data analysis showed our clinic reaching a majority male population, with 19 (73%) male patients and 7 (27%) female. Though this gap is in following with the previously demonstrated substantially male majority of PEH in NYC, it may also indicate a potential need for further outreach to female PEH by our clinic. Further analysis showed that 10 (38%) of our patients were uninsured, a greater proportion than the projected one-quarter provided by our community partners. Furthermore, 16 (62%) of our patients were determined to be Spanish-speaking, demonstrating a need for more Spanish-speaking care providers within our clinical practice.

## Conclusions

With our pilot interval and recent expansion to twice-monthly clinics complete, we look forward to gradually increasing our clinic frequency, patient volume, and depth of patient services. With preliminary indications of psychiatric needs in our community, we are currently working on assessing the feasibility of launching psychiatric care services in conjunction with our medical services. In addition, with interest from obstetrician/gynecologist (OB/GYN) providers in our health system, we look forward to conducting a thorough needs assessment within our patient population to evaluate gaps in OB/GYN care that we can address. We further aim to continue building robust sustainability within our clinic through continuous physician recruitment, residency education partnerships, expansion of hours of operation, and expansion to multiple sites in East Harlem. With respect to long-term vision, we continue to pursue the ultimate goal of developing a platform dedicated to serving PEH across our health system and NYC. We hope to play a role in establishing direct workflows between our clinic and discharge procedures for PEH at emergency departments in our health system, health system-wide consult services, respite care programs, inter-institutional free specialist care networks, and more.

It is clear that PEH face a vast multitude of barriers to accessing needed care in both acute and chronic settings. In NYC, these barriers are exacerbated due to highly complex housing landscapes and increases in housing instability across the city. Without medical safety-net interventions, PEH are at risk for continued worsening of both medical and housing-based outcomes. Here, we described the steps in which we approached targeting the growing disparities that PEH face at the intersection of health and housing through the creation of a community-based clinic. We are hopeful that, through documenting the process of launching our clinic, we can provide a reproducible framework for the development of similar programs to support PEH within NYC and across the United States.
